# Paediatric auditory brainstem implant: How we do it

**DOI:** 10.1007/s00701-025-06762-7

**Published:** 2026-01-19

**Authors:** Peter John Kullar, Simon Freeman, Scott Rutherford, Simon Lloyd, Martin O’Driscoll, Lise Henderson, Kerri Millward, Omar Pathmanaban

**Affiliations:** Manchester Skull Base Unit, Salford Royal Hospital, Stott Lane, Salford, UK

**Keywords:** paediatric auditory brainstem implant, hearing implants, hearing loss

## Abstract

**Background:**

The auditory brainstem implant (ABI) is a surgically implanted neuroprosthetic that may be used in the management of profound hearing loss in paediatric patients that are ineligible for cochlear implantation, primarily due to anatomical constraints.

**Method:**

We describe our technique for the implantation of a paediatric ABI. Surgical steps include post auricular incision, the creation of a periosteal flap, subperiosteal pocket and bony recess for the device, retrosigmoid craniotomy, placement of the electrodes adjacent to the cochlear nucleus and multi-layer closure. We highlight novel technical modifications to reduce the risk of complications and optimise outcomes.

**Conclusion:**

Paediatric auditory brainstem implants offer the possibility of sound awareness to patients with profound hearing loss that would not benefit from cochlear implantation or other hearing aid solutions.

**Supplementary Information:**

The online version contains supplementary material available at 10.1007/s00701-025-06762-7.

## Background and relevant surgical anatomy

The auditory brainstem implant (ABI) was developed in the late 1970 s by the House Ear Institute, initially for use in patients with profound hearing loss due to NF2 related schwannomatosis (NF2-SWN). Colletti et al. performed the first paediatric ABI surgery for auditory nerve aplasia in 2001 establishing the feasibility of this device in the paediatric population [1].

Three ABI systems are currently commercially available:

- Med El CONCERTO ABI implant system
- Oticon Digisonic® SP ABI brainstem implant
- Cochlear Nucleus ABI541

Each of these devices comprise an internal surgically implanted component and a magnetically attached external component. The external component of the device consists of a microphone, sound processor and transmitter. The internal component consists of a receiver-stimulator package, magnet and an electrode array and is surgically implanted into a recessed well in the squamous part of the temporal bone. The ABI electrodes are embedded in a polyester (Dacron) mesh and are implanted adjacent to the cochlear nucleus in the brainstem [2].

The auditory system is divided into the peripheral and central auditory system. The peripheral auditory system consists of the outer, middle and inner ear, housed within the temporal bone, and is the site of mechanotransduction whereby sounds is converted into electrical signals. The auditory nerve that conveys these signals from the cochlea to the cochlear nucleus in the brainstem is the first component of the central auditory system.

The cochlear nucleus complex comprises the dorsal and ventral cochlear nuclei situated on the dorsolateral side of the brainstem at the pontomedullary junction. The major input to the cochlear nuclei is the cochlear nerve, a component of the eighth cranial nerve (the vestibulocochlear nerve). The innervating axons maintain the tonoptopic organization that is established in the cochlea, with high frequencies mapped dorsally and low frequencies ventrally. Outputs from the cochlear nuclei include the superior olivary body, lateral lemniscus and inferior colliculus [5].

Intraoperative landmarks for the cochlear nucleus include [7]:

- The foramen of Luschka, the opening of the lateral recess of the fourth ventricle, which is indicated by the overlying choroid plexus and the flocculus of the cerebellum
- The 9th cranial nerve located at the caudal edge of the foramen of Luschka
- A straight vein running from the 9th/10th/11th cranial nerve complex to the 7th/8th cranial nerve complex forms the floor of the lateral recess
- A diminutive vein may be seen on the surface of the generally featureless area of brainstem indicating the position of the cochlear nucleus

## Description of the technique

We perform all non-NF2 paediatric ABIs using the retrosigmoid craniotomy. In contrast to the translabyrinthine approach, this requires a shorter operative time, avoids the space constraints of the paediatric mastoid and preserves the inner ear and vestibular apparatus [6]. The side to be implanted is selected based on a combination of radiological, audiometric and electrophysiological factors. If both ears display no functional hearing, then the side with the most accessible lateral recess based on imaging review is selected. If thresholds are found in one ear, but the child is not a candidate for a cochlear implant (CI), then this side should be selected based on the presumed superiority of the auditory cortex development.

### Positioning, neuromonitoring, incision and craniotomy

**Fig. 1:**
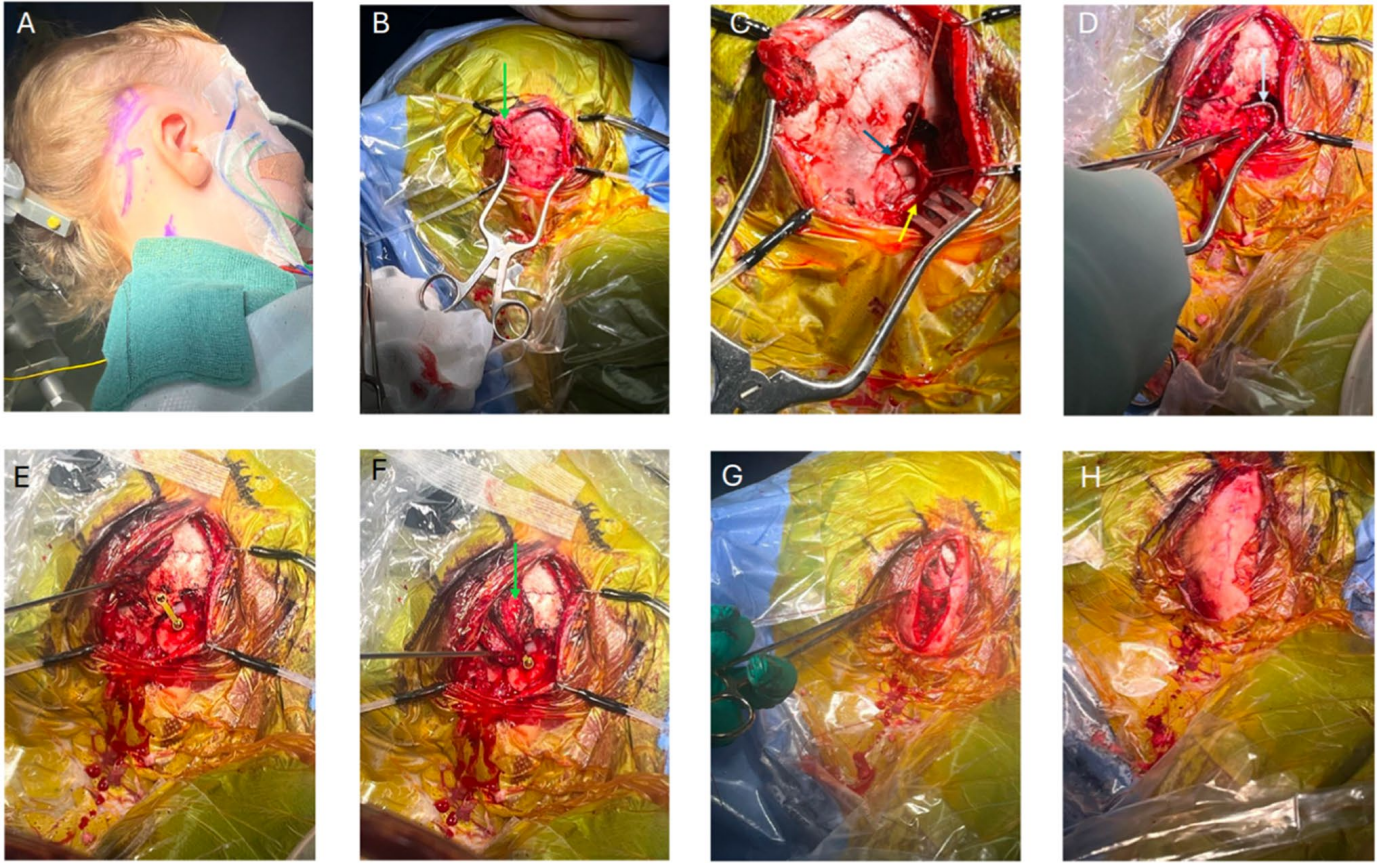
An overview of the retrosigmoid approach for paediatric ABI surgery. **A** The post auricular incision, site of craniotomy and subperiosteal pocket for the device are marked. **B** A superiorly based ‘U’ shaped flap (green arrow) is raised and the site for craniotomy is outlined. **C** Craniotomy is opened and pedicled inferiorly (yellow arrow), dural flaps (blue arrow) are secured with stay sutures. **D** The electrode paddle is sited into the lateral recess of the fourth ventricle and electrode lead (white arrow) connects to the receiver stimulator package placed in the bony recess. **E**,** F**,** G** The craniotomy is closed with a single dog bone miniplate and completely covered by reapposing the ‘U’ shaped periosteal flap. **H** The skin is closed in layers and a head bandage is then applied

**Fig. 2:**
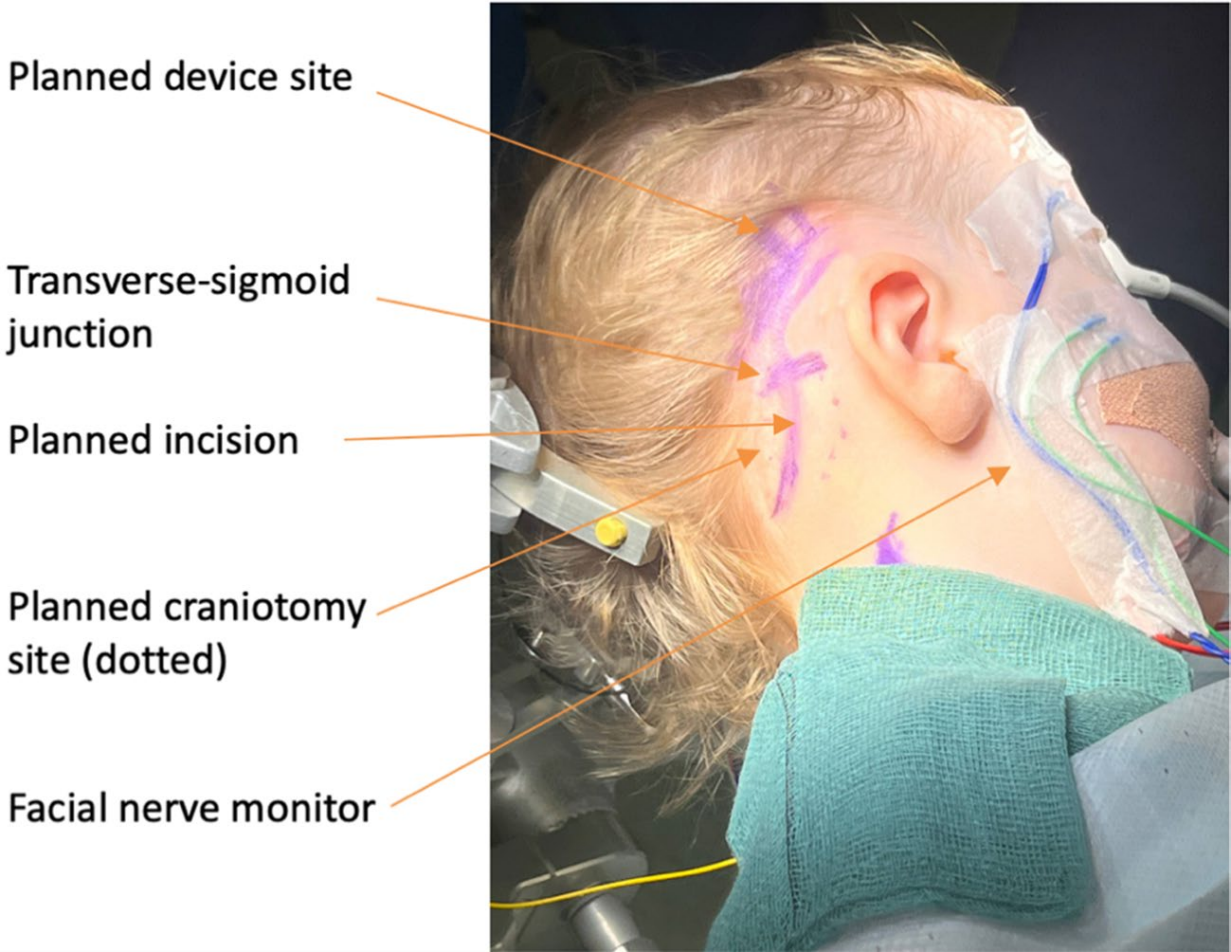
The post auricular incision, the site of craniotomy relative to the transverse-sigmoid junction and the proposed device site are marked. A facial nerve monitor with electrode sited in orbicularis oculi and orbicularis oris is used

**Fig. 3:**
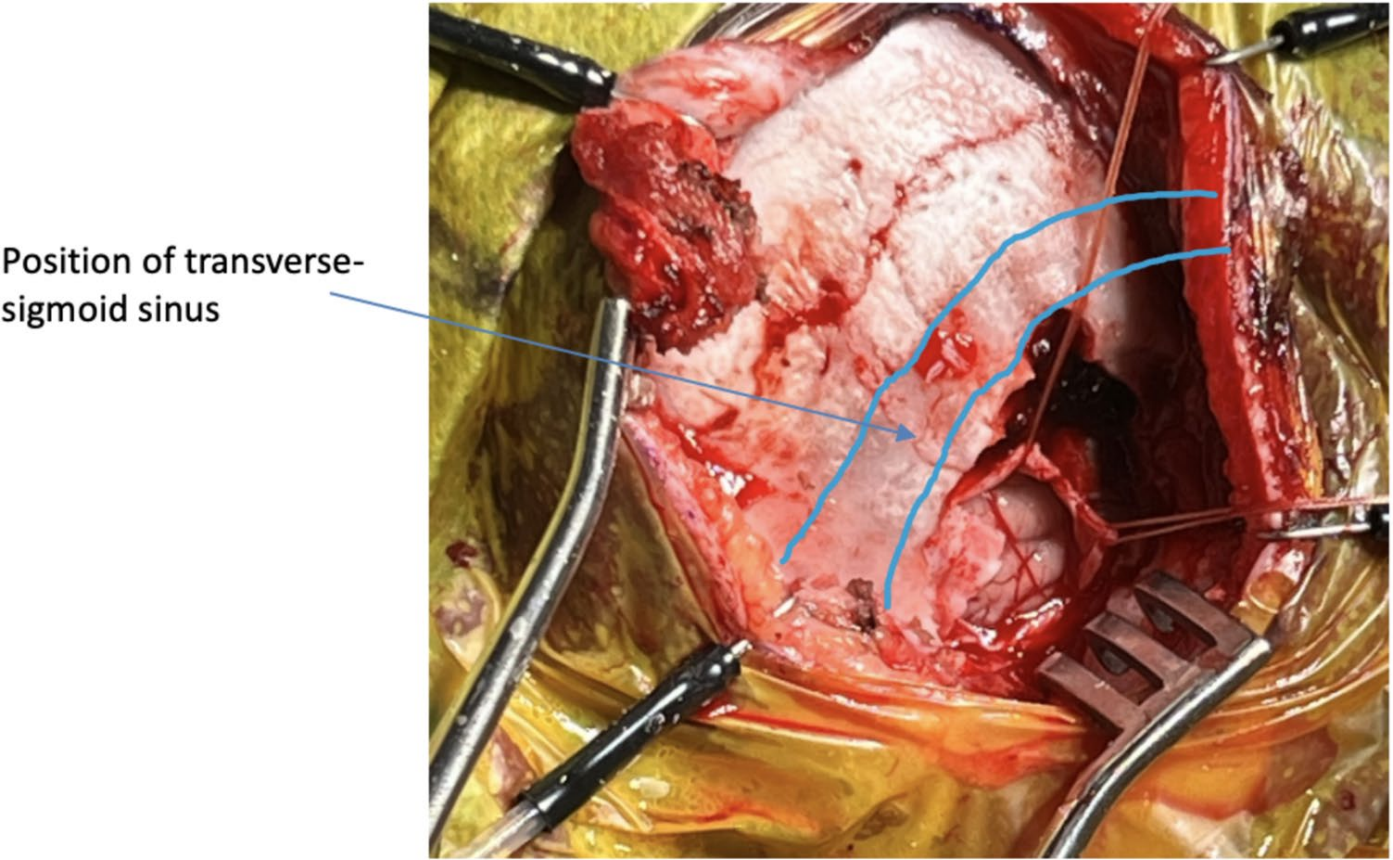
The position of the craniotomy relative to the sigmoid-transverse system. Dural flaps are retracted with stay sutures

The patient is placed into the supine position with head turned to the contralateral side with bolsters used to protect pressure areas. The Mayfield skull clamp is applied. Facial nerve monitoring is used, placing recording electrodes in the ipsilateral orbicularis oculi and orbicularis oris. Intraoperative implant evoked auditory brainstem response (EABR) is used to optimise the siting of the implant in the brainstem. EABR requires placement of recording electrodes at the midline, vertex, inion, over the C7 vertebrae and the contralateral mastoid. A curvilinear postauricular incision is performed elevating in a plane superficial to the mastoid/occipital periosteum and temporalis fascia (Fig. 1A and Fig. 2). A superiorly based ‘U’ shaped periosteal flap is then created followed by a subperiosteal pocket for the device underlying the area marked preoperatively on the skin (Fig. 1B). A 2 × 2.5 cm craniotomy is turned, bordered by the sigmoid sinus anteriorly and transverse sinus superiorly. The bone flap can then be ‘greensticked’ inferiorly leaving it pedicled to muscle at the inferior margin (Fig. 1C and Fig. 3).

### Surgical steps

**Fig. 4:**
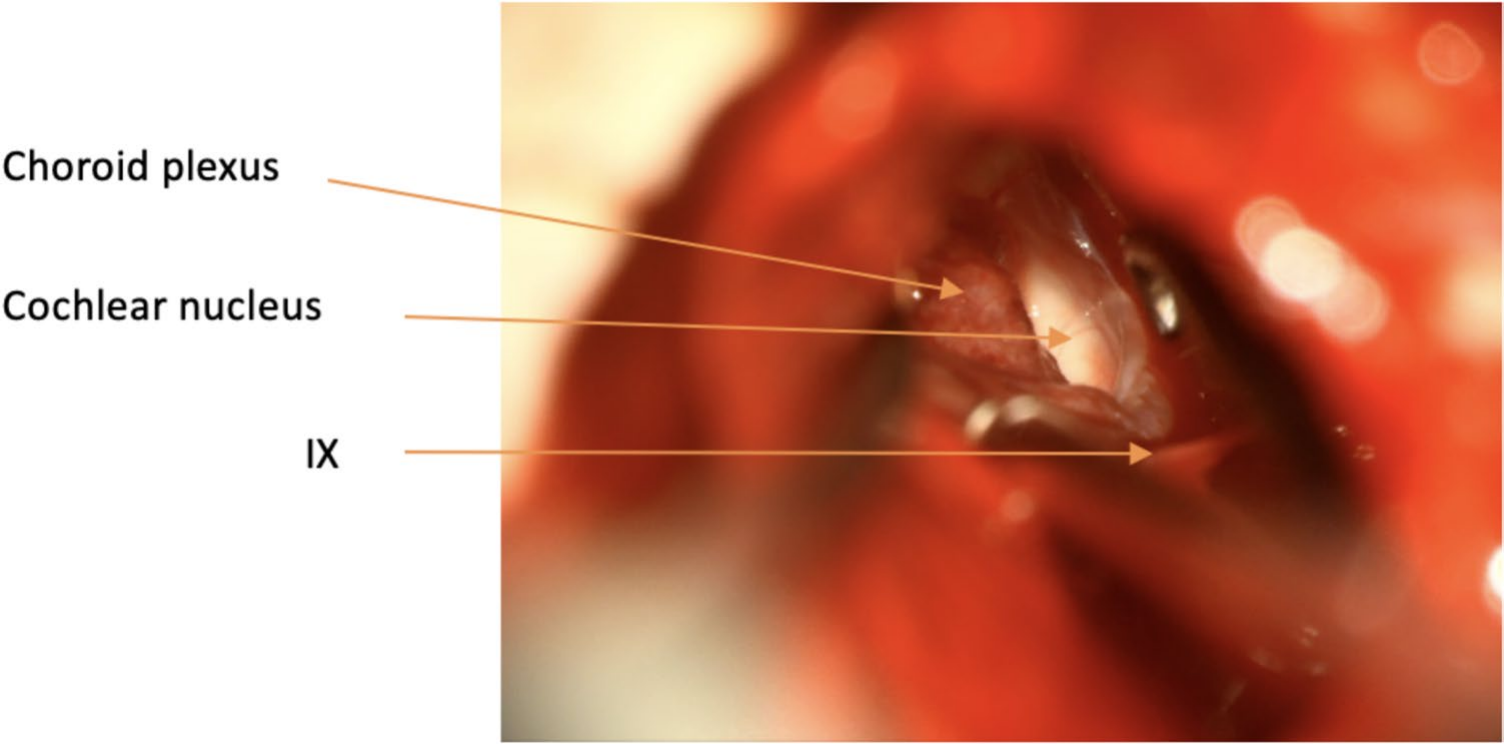
The lateral recess and cochlear nucleus are located using the 9th cranial nerves and choroid plexus as landmarks

The dura is opened in a Y-shape and the anterior and superior dural flaps are retracted with stay sutures (Fig. 3). The cerebellum is retracted posteriorly, and CSF is released from the cisterna magna. Using gentle retraction and arachnoid dissection the lateral recess is located using the 9th cranial nerves and choroid plexus as landmarks and its position marked with Surgicel (Ethicon, Cincinnati, USA) (Fig. 4).

A bony well for the receiver-stimulator package is then drilled into the squamous temporal bone using the silicone ABI template to determine the correct size. The well is positioned posterior and superior to the pinna. A bony channel is drilled from the bony recess to the craniotomy to accommodate the electrode lead.

**Fig. 5:**
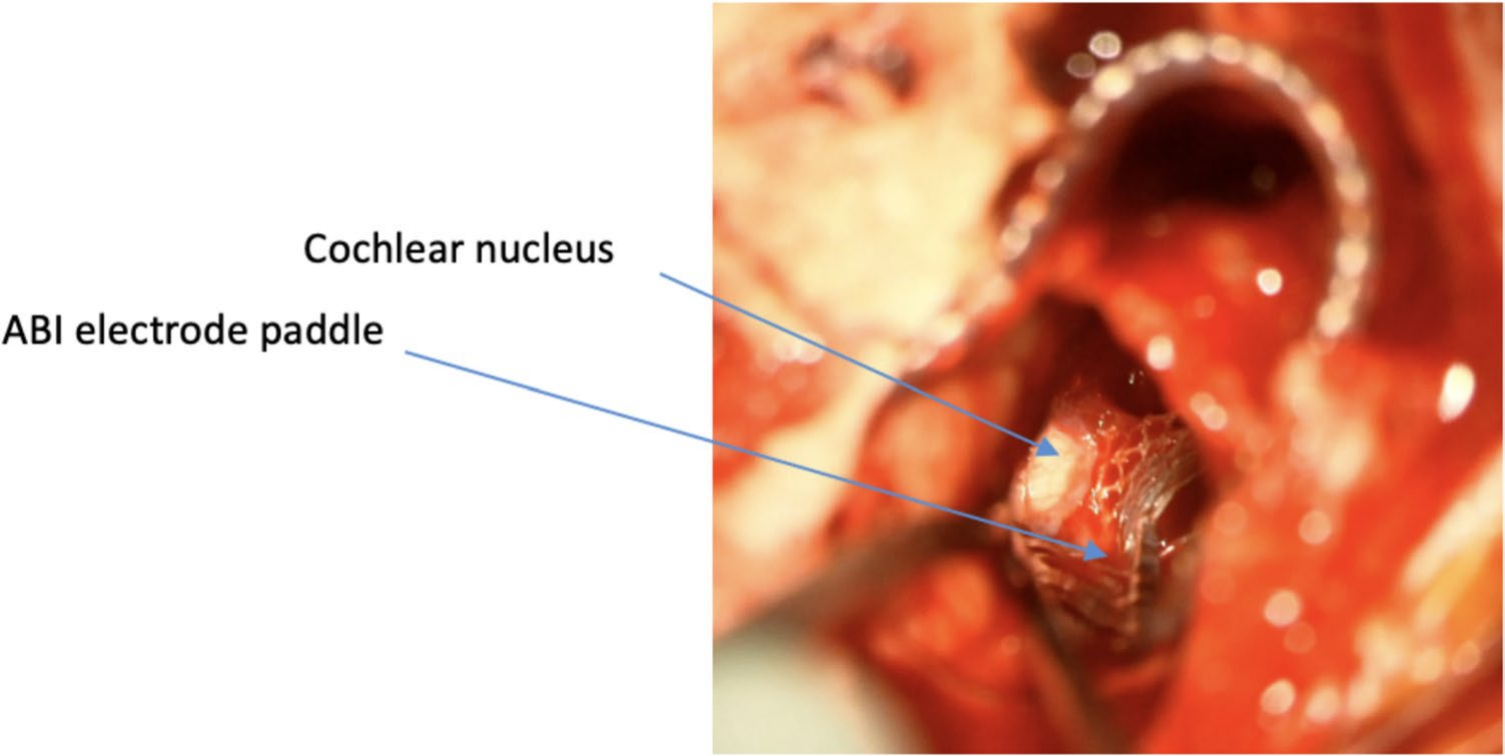
The ABI electrode paddle is placed into the lateral recess over the cochlear nucleus

The device can then be bought into the field and placed into the bony well and secured with Prolene (Ethicon, Cincinnati, USA) tie-down sutures. The tie-down sutures are anchored through holes drilled into the bone with a 1 mm diamond burr. The Dacron mesh of ABI electrode paddle is trimmed into an arrowhead. The mesh facilitates adherence to the brainstem. The paddle is then placed into the lateral recess over the cochlear nucleus that is located using the previously described intraoperative landmarks (Fig. 1D and Fig. 5). Neuronavigation is not routinely required.

Based on the EABR waveforms of individual electrodes, subtle adjustments can be made to the paddle position with reference to the paddle electrode map. Anaesthetic monitoring is required in case of non-auditory stimulation of the brainstem and subsequent cardiac arrhythmia. After placement, fibrin glue and a PTFE felt can be used to prevent paddle displacement (off licence usage).

The dura is tightly closed around the electrode lead using Duragen and fibrin glue. The dural closure cannot be completely watertight because the electrode lead must pass through it.

There is no space for an abdominal fat graft beneath the retrosigmoid craniotomy, however creating an additional incision for a small free fat graft to cover the lead’s dural exit point is not generally necessary.

The craniotomy bone flap is rotated into place and secured with a single dog-bone miniplate. The craniotomy site, electrode lead and device are completely covered by reapposition of the periosteal ‘U’ shaped flap (Fig. 1E, F, G). The skin incision is closed in layers and a mastoid pressure dressing is applied for 2 days (Fig. 1H). Patients are monitored in a paediatric intensive care unit for 24 h with hourly neurological observations. Length of stay is approximately 4 days. There is no routine postoperative imaging performed. Activation of the device with cardiac monitoring occurs after 6 weeks.

## Indications

NF2-SWN is a tumour predisposition syndrome characterized by bilateral vestibular schwannomas, that may cause progressive hearing loss with patients ultimately becoming profoundly hearing impaired either through the natural history of the tumours or treatment. The original indication for ABIs was for hearing restoration in patients with profound bilateral hearing loss as a complication of (NF2-SWN) [4]. Most adult ABI users are patients with NF2-SWN; however, this is rarely the case in paediatric ABI users who are mainly infants profound hearing loss and anatomy that precludes beneficial CI implantation.

In Europe, ABIs are also approved for patients over the age of 18 months who are unable to benefit from CI due to cochlear or retrocochlear abnormalities. Indications for paediatric ABI have been classified as definite and probable indicators [8]. Definite indicators include labyrinthine aplasia, cochlear aplasia and cochlear nerve aplasia whereas hypoplastic cochlea common cavity and hypoplastic cochlear nerve are probable indicators. MRI imaging of the inner ear and brain is a crucial step in assessing candidacy, while EABRs generated through transtympanic cochlear promontory stimulation may be used to evaluate the functional status of the cochlear nerve [3].

There is consensus opinion that the optimum age for implantation is between 18 and 24 months. This balances the requirement to implant at an age when brain plasticity is maximum with the low blood and CSF volumes in children under the age of 1 year [9].

## Limitations

Many ABI users benefit from improved environmental sound awareness and facilitated lip reading abilities. ABI outcomes in the non-tumour paediatric population are better than those implanted due to NF2-SWN. Rarely these patients can achieve closed or open set speech perception [10]. It should be noted that in our institution approximately 50% of implanted paediatric patients do not use their implant.

## How to avoid complications

The complication rate is low in experienced centres with CSF leak being the most common significant complication. To avoid this, we use a multilayer closure of the dura and skin incision. Our ‘U’ shaped periosteal flap also ensures the skin incision and periosteal incision do not overlap which reduces the chances of device infection requiring explantation in the case of skin breakdown. We perform a small craniotomy that is pedicled to the muscle inferiorly. This is not only faster than a traditional craniotomy but improves bone healing and reduces the chances of infection by improving vascular supply to the bone flap and negating the need for an inferior bone plate.

Other less frequent complications include cranial nerve palsies, pseudomeningocele, hydrocephaphlus, non-auditory stimulation and meningitis. We use tie down sutures to minimize the risk of device migration in the instance of CSF leak/pseudomengiocele. CSF leaks from the incision are managed on an individual basis but may include extra skin sutures and head wrap alone, or with a period of lumbar drainage. CSF nasal leaks are a rare occurrence as we do not open the middle ear. However, rarely it is possible if the mastoid is well pneumatised (unlikely in this age group) and air cells are opened doing the craniotomy. In this case we would wax off this air cells at the first operation and further rewaxing in case of a leak. In the case of non-auditory stimulation caused by inaccurate position of the electrodes or current spread within the brainstem, electrodes can be switched off or stimulation parameters altered.

## Specific information for the patient

It is critical the parents are counselled on the risks of ABI and these are balanced with the expected audiological outcomes for paediatric ABI insertion.

## 10 key point summary

1. Paediatric auditory brainstem implantation aims to restore hearing in children who are unable to benefit from traditional hearing aids or cochlear implants due to anatomic limitations such as cochlear nerve aplasia or cochlea malformations.
2. ABI is indicated in children with cochlear malformation or aplasia, cochlear nerve aplasia or NF2-SWN where there is no other hearing aid or implant solution.
3. ABI assessment and implantation is undertaken by a multi-disciplinary team including radiology, neurosurgery, neuro-otology and audiology.
4. Multi-modal assessment involving MRI and audiology is required to assess candidacy
5. A retrosigmoid craniotomy is performed and the cochlear nucleus located using the foramen of Luschka, 9th cranial nerve and the diminutive vein on the surface of the nucleus.
6. The ABI paddle electrodes are sited over the cochlear nucleus and EABR waveforms of individual electrodes are used to make subtle adjustments to the paddle position.
7. Tight dural closure and the periosteal flap reduce the risk of CSF leaks and wound infection.
8. Postoperative rehabilitation includes intensive auditory and speech therapy
9. Patients and their families should be counselled on the risks of brainstem implantation including infection, cerebrospinal fluid leak, device failure/no benefit from device and cranial neuropathies.
10. Ongoing research into electrode design, optogenetic stimulation, post-implantation programming and refinement to surgical techniques hold promise in optimising patient outcomes.

## Supplementary Information

Below is the link to the electronic supplementary material.

Supplementary Material 1 (MP4 272 MB)

## Data Availability

Not applicable.
